# The Impact of Treatments for Depression on the Dynamic Network Structure of Mental States: Two Randomized Controlled Trials

**DOI:** 10.1038/srep46523

**Published:** 2017-04-20

**Authors:** Evelien Snippe, Wolfgang Viechtbauer, Nicole Geschwind, Annelie Klippel, Peter de Jonge, Marieke Wichers

**Affiliations:** 1 University of Groningen, University Medical Center Groningen, Interdisciplinary Center Psychopathology and Emotion regulation, Groningen, the Netherlands; 2 Department of Psychiatry & Neuropsychology, School for Mental Health and Neuroscience, Maastricht University, Maastricht, the Netherlands; 3 Department of Clinical Psychological Science, Faculty of Psychology and Neuroscience, Maastricht University, Maastricht, the Netherlands; 4 Department of Neuroscience, Center for Contextual Psychiatry, KU Leuven, Leuven, Belgium; 5 Developmental Psychology, University of Groningen, Groningen, the Netherlands

## Abstract

Evidence is growing that vulnerability to depression may be characterized by strong negative feedback loops between mental states. It is unknown whether such dynamics between mental states can be altered by treatment. This study examined whether treatment with imipramine or treatment with Mindfulness-Based Cognitive Therapy (MBCT) reduces the connectivity within dynamic networks of mental states in individuals with depressive symptoms. In the Imipramine trial, individuals diagnosed with major depression were randomized to imipramine treatment or placebo-pill treatment (n = 50). In the Mind-Maastricht trial, individuals with residual depressive symptoms were randomized to Mindfulness-Based Cognitive Therapy (MBCT) or to a waiting-list control condition (n = 119). Lagged associations among mental states, as assessed with the Experience Sampling Method (ESM), were estimated at baseline and post-intervention. The results show that few of the dynamic network connections changed significantly over time and few of the changes after MBCT and imipramine treatment differed significantly from the control groups. The decrease in average node connectivity after MBCT did not differ from the decrease observed in the waiting-list control group. Our findings suggest that imipramine treatment and MBCT do not greatly change the dynamic network structure of mental states, even though they do reduce depressive symptomatology.

There have been many endeavors in psychiatric research to understand vulnerability to depression. A relatively recent theory is that mental disorders are the result of dynamic interactions between mental states that keep activating each other, as nodes in a network, and thereby eventually create a cluster of co-occurring symptoms which we can call a mental disorder^1^,^2^. This network perspective suggests that a mental state (e.g., feeling insecure) at one moment in time may trigger other mental states at a next moment in time (e.g., worry or a depressed mood), which may induce a cascade of negative mental states that reinforce each other^3^. Strong connections between momentary affect and cognitions (i.e., mental states) can thus be indicative of emotional inflexibility: the more a network of mental states informs itself, the less likely it is that internal or external factors can influence the emotion system^4^,^5^. Furthermore, increased connectivity (i.e., stronger links between current mental states and other mental states later in time) may reflect a more fragile architecture of the system in which sudden transitions to other, possibly worse, symptom states are more likely^6^.

Recent studies have used this network approach to gain insight into micro-level dynamics and how momentary mental states activate other mental states. Support is growing for the idea that the network structure of mental states is closely related to the vulnerability structure underlying psychopathology. For example, connections between momentary emotions have been shown to be stronger in individuals with depression in comparison with healthy controls^5^,^7^. Recently, it has also been shown that increasing connectivity in a network structure of mental states preceded a significant increase in depressive symptom levels over time within a single individual^8^.

Given the high relapse and recurrence rates after an initial episode of major depression^9^,^10^, there is a need to examine not only how *symptoms* can be reduced, but also how *underlying vulnerability* to depression can be diminished. It would therefore be valuable to examine whether treatments for depression can not only achieve reductions in symptom levels, but can actually change the dynamic interactions between mental states associated with depression.

To examine whether treatment reduces the negative dynamics between mental states associated with depression, it is important to assess the moment-to-moment associations between negative and positive mental states within individuals^11^. A cross-sectional network approach, using a single measurement per individual, can only examine whether symptoms co-occur across individuals and not how mental states follow each other over time^12^. Therefore, frequent prospective assessments of mental states are needed because they allow an examination of the temporal associations (i.e., from one moment to the next moment in time) between mental states *within* individuals. Furthermore, to examine whether treatment alters the connections within a network of mental states, we can test whether the dynamic network structure changes over time and whether this differs between treatment groups using permutation procedures that were recently developed for this purpose.

To our knowledge, the current study is the first to construct network structures with experience sampling (ESM) assessments^13^ before and after treatment and that examines changes therein. The aim of the study is to examine whether a pharmacological and a psychological treatment can reduce connectivity within a dynamic network structure of mental states (i.e., feeling down, agitated, insecure, worried, and cheerful) in individuals with depressive symptoms. We tested this hypothesis in two unique randomized controlled trials, one of which examined the effectiveness of treatment with imipramine versus placebo (Imipramine trial) while the other examined mindfulness-based cognitive therapy (MBCT) versus a waiting-list control condition (Mind-Maastricht trial). We aimed to compare change in specific dynamic associations as well as overall connectivity within the two trials; thus, between the imipramine condition and the placebo condition on the one hand, and between MBCT and the waiting-list control condition on the other hand.

## Results

### Characteristics of the study sample

**Figure 1:**
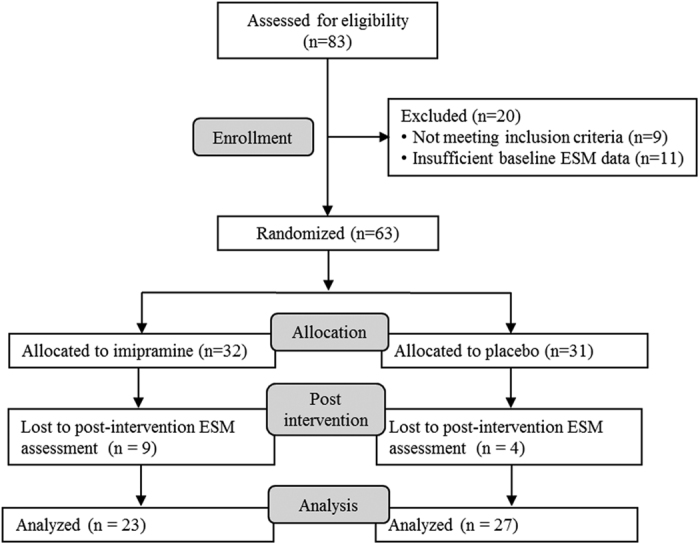
Flow chart of participants of the Imipramine trial included in the current study.

**Figure 2:**
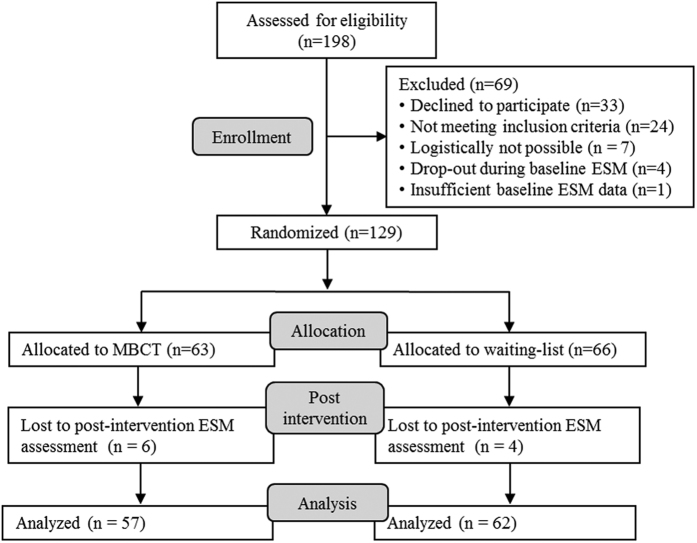
Flow chart of participants of the Mind-Maastricht trial included in the current study.

The flow of participants in the two studies is presented in Figs 1 and 2. In the Imipramine trial, 49 of the 63 randomized participants completed 6 weeks of treatment (for details, see previous papers^14^,^15^) and 50 of the 63 randomized participants provided ESM data at baseline and post-intervention and thus were included in the present analyses. In the Mind-Maastricht trial, 3 of the 130 randomized participants discontinued study participation (for details, see previous paper^16^) and 119 of the 130 randomized participants provided ESM data at baseline and post-intervention and were included in the analyses. Participants in the MBCT group reported practicing for 30.3 minutes (*SD* = 11.2) per day on average. Mindfulness practice was calculated as minutes spent on long formal exercises. In addition to the formal meditation exercises, participants reported to have engaged in informal meditation exercises (such as the 3-minute breathing space) 2.2 times a day on average (*SD* = 1.0).

In the Imipramine trial, the mean age of the participants was 42.5 (SD = 9.1), the majority of the sample was female (74%) and married or cohabiting (80%). In the Mind-Maastricht trial, the mean age was 44.0 (SD = 9.7), the majority of the participants were female (76%) and married or cohabiting (62%).

### Change in mental state levels and depressive symptom levels over time

In the Imipramine trial, both treatment with imipramine and placebo was associated with significant improvements in depressive symptoms and mental states (i.e., down, agitated, worried, and cheerful) from baseline to post-intervention (see Table 1). Only feeling insecure did not significantly change after treatment with placebo. The imipramine and placebo groups did not differ significantly in the amount of change in mental states over time, whereas imipramine was associated with a larger reduction in depressive symptoms than placebo.

In the Mind-Maastricht trial, MBCT was associated with significant improvements in depressive symptoms and all of the mental states from baseline to post-intervention (see Table 1, also reported before^16^). Individuals who received MBCT showed significantly larger improvements in both depression and mental states than the waiting-list control condition. Only the reduction in feeling agitated over time did not differ significantly between the MBCT and control groups.

### Change in specific network connections over time

**Figure 3:**
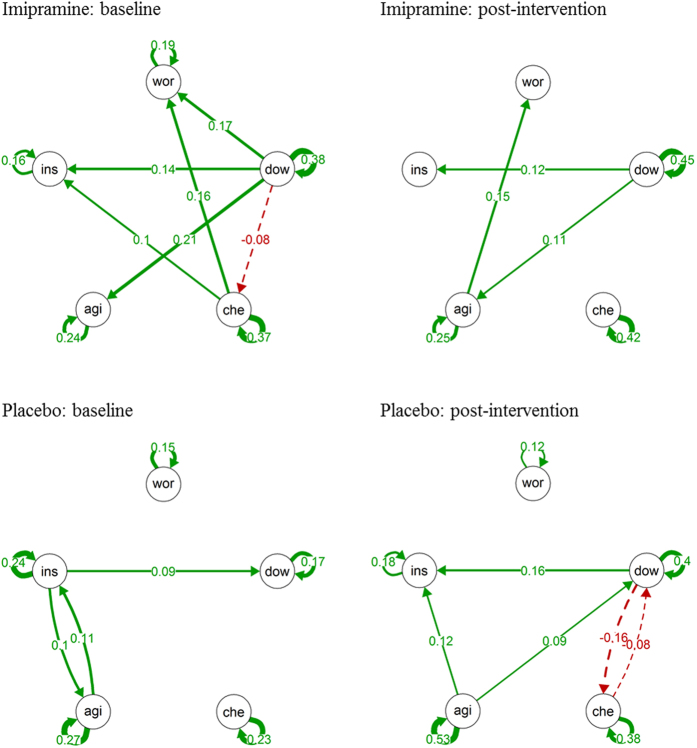
Networks of momentary mental states in the Imipramine Trial. *Note*. Networks of momentary mental states in the Imipramine Trial. wor = worried, dow = down, che = cheerful, agi = agitated, ins = insecure. The arrows represent the significant lagged associations between the mental states. Solid arrows reflect positive associations and dashed arrows reflect negative associations. The thickness of the lines reflect the strength of the associations; the thicker the line, the stronger the association.

**Figure 4:**
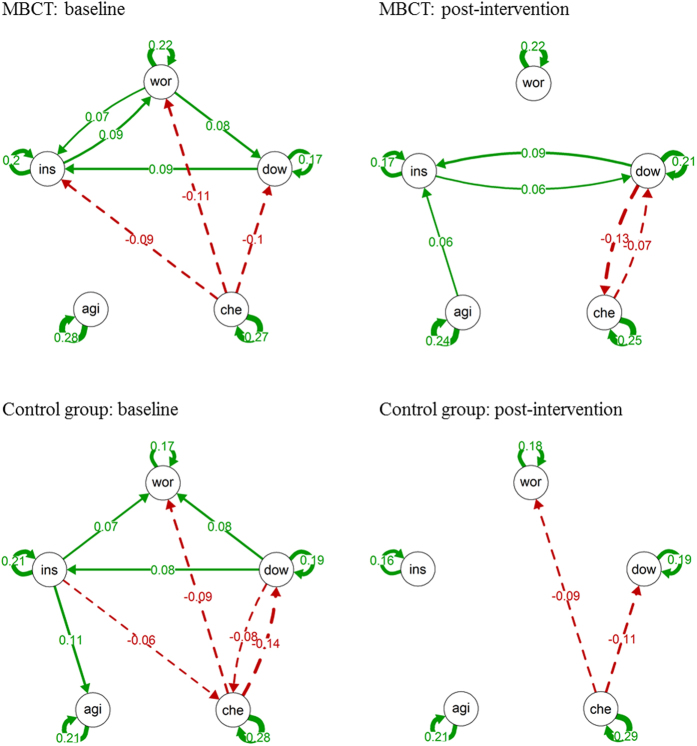
Networks of momentary mental states in the Mind-Maastricht Trial. *Note*. wor = worried, dow = down, che = cheerful, agi = agitated, ins = insecure. The arrows represent the significant lagged associations between the mental states. Solid arrows reflect positive associations and dashed arrows reflect negative associations. The thickness of the lines reflect the strength of the associations; the thicker the line, the stronger the association.

The dynamic networks of mental states at baseline and post-intervention are presented in Figs 3 and 4. In the group receiving imipramine, none of the network connections changed significantly from baseline to post-intervention (see Table 2). This indicates that the time-lagged associations among the mental states from one moment to the next moment in time did not change after treatment with imipramine. In the placebo group, there was a significant increase in the autocorrelation (i.e., the time-lagged effect of a mental state on itself) of feeling down and feeling agitated from baseline to post-intervention. There was also a significant increase in the positive effect of feeling down on feeling insecure over time in the placebo group, which was significantly larger than the change over time found in the imipramine group. Furthermore, there was a significant difference between the two groups in the change in the effect of feeling down on feeling cheerful, which non-significantly increased in the placebo group and non-significantly decreased in the imipramine group. None of the other baseline to post-intervention changes in network connections differed significantly between the imipramine and the placebo group.

In the Mind-Maastricht trial, there was a significant decrease in the effect of feeling worried on feeling down in the MBCT group and a significant decrease in the effect of feeling down on feeling insecure in the waiting-list control condition (see Table 3). These changes did not differ significantly between the two groups. Change in only one of the network connections over time differed significantly between the MBCT and the waiting-list control group: whereas the negative association between feeling down and cheerful became stronger over time in MBCT, it became weaker over time in the waiting-list control group.

### Change in overall network connectivity

Next, we examined the change in average node connectivity (i.e., the average of the absolute strength of the lagged associations between the mental states within a network) from baseline to post-intervention. In the Imipramine trial, there was no significant change in average node connectivity over time in the imipramine group (change = −0.01, *p* = 0.40) whereas the average node connectivity *increased* over time in the placebo group by 0.04 (*p* < 0.01). The change in overall connectivity differed significantly between the imipramine and placebo group (difference = −0.05, *p* = 0.01). In the Mind-Maastricht trial, the average node connectivity decreased by 0.02 over time both in the group receiving MBCT (*p* = 0.06) and the waiting-list control condition (*p* = 0.03). The change in overall connectivity did not significantly differ between MBCT and the waiting-list control condition (*p* = 0.95).

## Discussion

We set out with the aim to examine whether a psychological treatment (i.e., MBCT) and a pharmacological treatment (i.e., imipramine) can alter the dynamic network structure of mental states (i.e., feeling worried, down, insecure, cheerful, and agitated) in individuals with depressive symptoms. The study was performed in data from two unique randomized controlled trials, with momentary assessments of mental states before and after treatment. These repeated assessments allowed us to gain insight in the dynamic associations among mental states within individuals, as opposed to studies of cross-sectional symptom networks^12^. To our knowledge, this study is the first to examine the effect of treatment on changes in longitudinal dynamic networks of mental states.

The findings of the study do not support that MBCT and imipramine impact on the dynamic associations within a network of mental states associated with depression. Only two of the baseline to post-intervention changes in connections differed significantly between the imipramine and placebo and between the MBCT and waiting-list control group. Change in average node connectivity (i.e., a measure of overall network connectivity) over time also did not differ significantly between the MBCT and waiting-list control group. Although change in average node connectivity did significantly differ between the imipramine and placebo group, this change does not seem to be induced by imipramine, since change over time in the imipramine group was not significant. In sum, it seems most parsimonious to conclude that MBCT and treatment with imipramine were not able to impact greatly on the dynamic associations within a network of momentary mental states.

The finding that MBCT and imipramine were not effective in changing the dynamic network structure of mental states is especially remarkable since MBCT and imipramine *were* effective in reducing depressive symptoms in comparison with the control groups. Change in the average levels of mental states was also larger in the MBCT group in comparison with the waiting-list control condition. This may indicate that mental states themselves (i.e., the nodes in the network) can change independently from network connections between these mental states. Second, the findings suggest that even when depressive symptoms decrease, underlying vulnerability, as indexed by network connections among the mental states, can remain present. Thereby, the study findings may offer an explanation for why many individuals remain vulnerable to the experience of a relapse or recurrence of depression after successful treatment of their symptoms^17^. The fact that it may be hard to change vulnerability underlying depression has also been reported by previous studies showing that pharmacological and psychological treatments do not always decrease other indicators of vulnerability for depression, such as neuroticism^18^, stress reactivity^19^, and cognitive reactivity^20^. On the other hand, it has also been shown that MBCT is efficacious in reducing depressive relapse and recurrence^21^,^22^. Based on this finding, one could also speculate that our assumptions based on the network approach do not hold, i.e., that a decrease in the strength of the connections within a dynamic network of mental states reflects a decrease in underlying emotional vulnerability. However, several studies support the assumption underlying the network approach, showing that dynamic network connections are stronger in individuals with psychopathology than in those without^5^,^7^ and that an increase in the strength of the dynamic associations among mental states occurs right before a transition to a depressed state^8^.

In contrast to the finding that *treatment* did not impact on average node connectivity, we found that *time* did. Over time, average node connectivity decreased both in the MBCT group as well as in the waiting-list control group, although the change in the MBCT group was just not significant. Thus, change in overall network connectivity is possible, as was also indicated in a previous study^8^. This decrease in overall network connectivity may reflect reduced emotional vulnerability to depression over time, although this change does not seem to be driven by treatment. In addition, we showed that average node connectivity *increased* in the placebo group, which is remarkable since depressive symptoms and negative mental states did decrease in this group.

An explanation for the fact that treatment did not impact on connectivity within networks of mental states might be that treatment decreased *different* network connections in each individual. For example, MBCT might reduce the effect of feeling down on worrying for one person while it may decrease the effect of feeling insecure on feeling agitated for another person. An important avenue for future research is therefore to examine individual differences in the effect of treatment on underlying vulnerability structures using network models that are estimated for each individual separately. Such an approach may in fact guide personalized clinical decision making. This underscores the potential relevance of the current network approach for personalized medicine and the need for further validation thereof using time-series analyses and replicated single-case designs.

An alternative explanation for our findings is that we were not able to detect significant changes over time and group differences between the networks because of power issues. This explanation, however, seems unlikely as more significant effects were reported in the smaller Imipramine trial than in the larger Mind-Maastricht trial.

Although this study was not designed to make comparisons *between* the trials, the difference in severity of depressive symptoms between the Imipramine trial and the Mind-Maastricht trial could be a limitation of the study. Whereas participants in the Imipramine trial had a current diagnosis of major depression, participants in the Mind-Maastricht trial had a history of depression and residual depressive symptoms. Although one could argue that the low levels of depressive symptoms explain why specific network connections did not change in MBCT, we do not think this is very plausible since we examined change in vulnerability underlying depression, which may be present in the absence of severe symptoms. In addition, we cannot exclude the possibility that MBCT and imipramine affected dynamic network connectivity later in time, since we do not have follow-up ESM assessments of mental states in both trials. Furthermore, although only study completers were included in the current study, data on medication adherence was not available, which might have affected the findings. Finally, future studies could consider including a broader variety of positive emotions, as the current study included mainly negative mental states.

It can be tentatively concluded that imipramine treatment and MBCT do not strongly impact on connectivity within networks of mental states. The findings highlight that underlying emotional dynamic structures might be hard to change by treatment in individuals who have been depressed. It is possible that other treatments are more effective in changing the negative feedback loops between negative and positive mental states. Besides research on other types of treatment, we suggest that future studies estimate dynamic networks for each individual separately to capture individual differences. These person-specific networks may be used to identify specific subgroups of individuals with similar changes in dynamic associations among mental states.

## Method

### Study design

The study is a secondary analysis of two previously performed randomized clinical trials that were conducted independent of each other. In both trials, ESM was performed before and after the intervention period. Study 1 (Imipramine trial) concerns a double-blind placebo-controlled trial comparing the effects of imipramine and placebo in patients with a diagnosis of current major depression^14^. Study 2 (Mind-Maastricht trial) concerns a randomized controlled trial comparing the effects of Mindfulness-Based Cognitive Therapy (MBCT) and a waiting-list control condition in patients with a history of depression and current residual depressive symptoms^16^. Both studies were approved by the Medical Ethics Committee (MEC) of Maastricht University and were performed in accordance with the ethical standards of the MEC and the Declaration of Helsinki. Specific details on the designs, participants, and procedures of both studies are described elsewhere^14^,^16^.

### Participants

All participants provided written informed consent for study participation. The inclusion criteria of the Imipramine trial and the Mind-Maastricht trial differed from each other as both trials were conducted independently of each other. The inclusion criteria of the Imipramine trial were: age between 18 and 65 years, a DSM-IV diagnosis of current major depressive disorder, a score of ≥18 on the 17-item Hamilton Depression Rating Scale (HDRS)^23^, a score of ≥4 on the Clinical Global Impressions Scale (CGI), no current use of psychotropic medications, and no diagnosis of a major medical disorder. In the original trial, 63 patients with a diagnosis of major depressive disorder were recruited from primary care practices. In the current study, participants who had completed ESM assessments both at baseline and after treatment with imipramine (n = 23) or placebo (n = 27) were included in the analyses.

The inclusion criteria of the Mind-Maastricht trial were: a score of ≥7 on the 17-item Hamilton Depression Rating Scale (HDRS), no diagnosis of current major depression, schizophrenia, or psychotic episodes in the past year, and no recent changes or upcoming changes in ongoing psychological or pharmacological treatment. In total, 130 patients were recruited from outpatient mental health care practices and advertisements. Analyses of the current study included patients with ESM assessments at baseline and after MBCT (n = 57) or after the waiting period (n = 62).

### Interventions

Participants in the Imipramine trial received either twice daily imipramine (200 mg per day) or placebo (4 capsules per day) for 6 weeks. In case of intolerance, the dose was decreased to either 100 mg of imipramine per day or 2 placebo capsules per day. Both patients and the assessors of the primary outcomes were blinded to the type of treatment patients received. After 6 weeks, the treatment could be prolonged to 18 weeks.

Patients in the Mind-Maastricht trial were randomized to either an 8-week MBCT group program or to an 8-week waiting-list control condition. After the post-intervention assessment, participants in the control condition had the opportunity to take part in an MBCT program. MBCT consisted of eight weekly group sessions of 2.5 hr and homework assignments of 30 to 60 minutes a day. The MBCT sessions followed the MBCT guidelines of Segal and colleagues^24^. The sessions covered meditation exercises (e.g., body-scan, sitting meditation), yoga exercises, and discussion of the exercises and homework assignments.

### Experience Sampling Methodology (ESM)

In both studies, participants completed ESM assessments at baseline and after the intervention period (i.e., after 6 weeks of imipramine or placebo and after 8 weeks of MBCT or the waitlist period). All participants received a detailed one-on-one explanation of the experience sampling procedure before the baseline assessment. With ESM, individuals assess momentary affect, behavior, and context repeatedly during daily life. ESM allows the assessment of daily life behaviors and affect in a prospective and ecologically valid way while minimizing retrospective bias^13^. Participants completed ESM reports in response to a signal emitted by a programmed wristwatch at semi-random moments in ten 90-minute time blocks between 7:30 am and 10:30 pm. ESM reports were completed on 6 consecutive days, resulting in a maximum of 60 observations at each assessment period per person.

### ESM Measures

First, we selected mental states associated with depression that (1) were assessed in both the Imipramine trial and the Mind-Maastricht trial, and (2) represent different underlying constructs, including negative affect (high and low arousal), positive affect, and worry. The candidate mental states were the items “down”, “tired”, “insecure”, “anxious”, “agitated”, “worried”, “satisfied”, “cheerful”, “enthusiastic”, and “relaxed”. From theses candidate items, five mental states were selected that were not too highly correlated concurrently (*r* < 0.55 among the deviations from the person mean of each item), and showed considerable within-person variation over time (Mean Squared Successive Difference (MSSD) > 0.90). The final selection comprised of the items “down”, “agitated”, “insecure”, “worried”, and “cheerful”. These ESM adjectives were rated on 7-point Likert scales ranging from 1 (*not at all*) to 7 (*very*).

### Depressive symptoms

Severity of depression was assessed with the 17-item Hamilton Depression Rating Scale (HDRS)^23^ in both trials. The HDRS was administered at baseline and after treatment or the waiting-list control period (post-intervention). The HDRS ranges from no depressive symptoms (0) to severe depressive symptoms (52).

### Statistical Analyses

#### Symptom change over time

Changes in mental state levels from baseline to post-intervention were examined with multilevel analyses with ESM observations nested within individuals. In a first set of models, time (0 = baseline, 1 = post-intervention) was included as a fixed effect and an ESM variable (i.e., down, agitated, insecure, worried, or cheerful) was included as the outcome variable. These models were run for all treatment conditions separately. In a second set of models, time and treatment condition (Imipramine trial: 0 = placebo, 1 = imipramine; Mind-Maastricht trial: 0 = control group, 1 = MBCT), as well as their interaction were included as fixed effects. These models included random intercepts at the subject-level and random slopes for time.

Changes in depressive symptoms levels (HDRS scores) over time were examined with paired t-tests for each treatment condition separately. Differences between the treatment conditions in the change in HDRS scores over time were tested with independent samples t-tests.

#### Change in dynamic networks over time

To construct the dynamic networks of mental states, we used the regression coefficients of the time-lagged associations among the mental states that were computed using autoregressive multilevel modeling^25^. In the multilevel autoregressive analyses, it was tested whether an ESM variable X at each time point (t) could be predicted by lagged values of itself (reflecting autocorrelation) and the other ESM variables (t-1), as has been done in previous studies using intensive longitudinal data^26^,^27^. Deviations from the person mean of the ESM variables were used as predictor variables, to separate within-subject effects from between-subject effects^28^. In addition, to model any trend in a particular outcome (i.e., ESM variable) over the entire observation period, a time variable (i.e., the observation number) was included as predictor in the models. All multilevel models included random intercepts and random slopes for the time variable (which were allowed to be correlated).

For each ESM variable, a separate multilevel analysis was performed at each phase (i.e., baseline and at post-intervention) and treatment condition. Based on the estimated regression coefficients (fixed effects) of these models, one network (describing the time-lagged associations among the mental states) was constructed per phase and treatment condition, resulting in eight networks of mental states (four conditions with each a network at baseline and post-intervention). Visualizations of the networks were made using the *qgraph* package in R^29^.

#### Model testing

We used permutation testing procedures^30^ rather than standard test procedures, as permutation testing can solve some of the statistical issues that we encountered in the analyses (see online supplementary information for R scripts). In particular, it was not possible to include random slopes for the time-lagged ESM variables in the multilevel analyses as this would result in a large number of parameters in the models which led to model converge problems. As a result, the standard errors of the fixed effects, and therefore also the p-values, do not account for the between-subject variability in the slopes. Permutation testing does not require parametric assumptions about the null distribution of test statistics to generate p-values, but computes the p-values by comparing the results obtained under repeated permutations of the data against the results obtained using the actually observed data. Therefore, with this procedure, the fact that random slopes are not included in the model will not affect the estimation of the p-values.

First, we tested the significance of the lagged associations per treatment phase and condition. Here, the distribution of the coefficients under the null hypothesis can be generated by permuting the values of the outcome variable within subjects and then refitting the autoregressive multilevel model. Repeating this process a large number of times, the permutation-based p-value for a coefficient is then given by the proportion of cases where the coefficient under the permuted data was as extreme or more extreme than the value of the coefficient observed under the actual data (multiplied by 2 for a two-tailed test). The results of this first step does not concern our primary hypotheses but is described to provide the reader with all necessary information on the effects.

Second, we tested the change in the lagged associations from baseline to post-intervention for each treatment condition. Here, the phase variable is permuted within subjects (so that post-intervention data of a subject may be treated as baseline data and vice-versa) and the multilevel autoregressive model for a particular outcome is then fitted to the synthetic ‘baseline’ and ‘post-intervention’ data, based on which we computed the change in each coefficient.

Third, we tested whether the change in the lagged associations from baseline to post-intervention differed significantly between treatment conditions. Here, the group variable is permuted (essentially mirroring the random assignment of subjects to the two conditions) and the multilevel autoregressive model is fitted to each synthetic ‘group’ at each time point. Finally, we tested the change in the average node connectivity (representing overall connectivity) within the networks from baseline to post-intervention within groups and whether the change differed between groups. To do so, we calculated the average of the absolute strength of all the network connections^31^,^32^ (i.e., the absolute values of the regression coefficients) within the networks at baseline and post-intervention. We then computed the amount of change in the average node connectivity from baseline to post-intervention for each treatment condition and the difference in the amount of change between groups. The permutation-based p-values are then generated as described above, either permuting the time variable or the group variable, depending on the hypothesis tested.

Ideally, exact p-values would be computed by generating all possible permutations. However, the number of possible permutations is too large to enumerate for these data (e.g., for the 50 patients in the Imipramine trial, there would be 2^23^ or 2^27^ possible permutations of the time variable depending on the group and 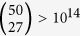 possible permutations of the group variable). Instead, we approximate the exact p-values by considering a large number (i.e., 100,000) of random permutations (also called Monte Carlo sampling)^33^, which guarantees a standard error of <0.002 in the estimates of the exact p-values.

The permutation-based tests were conducted on a cluster computer with 128 cores (8 AMD Opteron Processor 6276 CPUs each with 16 cores) and 512 GB of RAM using parallel/multicore processing (total computation time for the analyses was approximately 500 core hours). Analyses were run in R 3.2.3^34^, using the *nlme* package^35^ for fitting the multilevel models.

## Additional Information

**How to cite this article**: Snippe, E. *et al*. The Impact of Treatments for Depression on the Dynamic Network Structure of Mental States: Two Randomized Controlled Trials. *Sci. Rep.* **7**, 46523; doi: 10.1038/srep46523 (2017).

**Publisher's note:** Springer Nature remains neutral with regard to jurisdictional claims in published maps and institutional affiliations.

## Supplementary Material

Supplementary Information

## Figures and Tables

**Table 1:** Means and standard deviations of the mental states at baseline and post-intervention.

Variable	Imipramine (*N* = 23)		Placebo (*N* = 27)		MBCT (*N* = 57)		Control (*N* = 62)	
	Base	Post	Base	Post	Base	Post	Base	Post
Down	2.5 (1.9)	1.9 (1.3)**	3.0 (1.7)	2.1 (1.4)**	2.4 (1.6)	1.8 (1.3) **^,††^	2.5 (1.6)	2.4 (1.5)
Agitated	2.7 (1.8)	2.1 (1.5)*	3.3 (1.9)	2.2 (1.6)**	2.6 (1.8)	2.1 (1.5) **	2.9 (1.8)	2.6 (1.6) **
Insecure	2.1 (1.4)	1.8 (1.1)*	2.7 (1.9)	2.1 (1.5)	2.6 (1.7)	2.2 (1.5) **^,††^	2.6 (1.6)	2.6 (1.6)
Cheerful	1.7 (1.2)	2.6 (1.6)**	2.2 (1.4)	3.0 (1.7)**	3.8 (1.6)	4.4 (1.5) **^,††^	4.1 (1.5)	4.0 (1.6)
Worried	1.6 (1.2)	1.3 (0.9)**	2.0 (1.7)	1.5 (1.2)**	2.9 (1.9)	2.0 (1.5) **^,††^	2.8 (1.8)	2.7 (1.7)
HDRS	24.7 (4.2)	8.5 (6.3)**^,†^	23.8 (2.8)	12.7 (6.3)**	10.4 (3.6)	7.1 (4.7)**^,††^	10.0 (3.5)	9.4 (4.0)

Note. Base = baseline, post = post-intervention, *p < 0.05 (effect of time), **p < 0.01 (effect of time), ^†^p < 0.05 (effect of time × treatment), ^††^p < 0.01 (effect of time × treatment).

**Table 2:** Imipramine Trial: Change in associations between mental states over time.

	Imipramine			Placebo			Differ
	Base	Post	∆	Base	Post	∆	∆
Down_t−1_- Down_t_	0.38**	0.45**	0.07	0.17**	0.40**	0.22*	−0.15
Agitated_t−1_- Down_t_	0.06	0.08	0.02	0.06	0.09*	0.03	−0.01
Insecure_t−1_- Down_t_	0.01	−0.01	−0.02	0.09*	0.10	0.01	−0.04
Worried_t−1_- Down_t_	0.00	0.03	0.02	0.05	−0.04	−0.08	0.11
Cheerful_t−1_- Down_t_	−0.06	−0.01	0.05	−0.00	−0.08*	−0.08	0.12
Down_t−1_- Agitated_t_	0.21**	0.11*	−0.10	0.03	0.03	−0.00	−0.09
Agitated_t−1_- Agitated_t_	0.24**	0.25**	0.01	0.27**	0.53**	0.27**	−0.25
Insecure_t−1_- Agitated_t_	0.03	−0.03	−0.07	0.10*	0.02	−0.08	0.02
Worried_t−1_- Agitated_t_	0.03	0.04	0.02	−0.01	−0.07	−0.07	0.08
Cheerful_t−1_- Agitated_t_	−0.04	−0.00	0.04	0.02	−0.04	−0.06	0.10
Down_t−1_- Insecure_t_	0.14*	0.12**	−0.01	−0.05	0.16**	0.21**	−0.23*
Agitated_t−1_- Insecure_t_	0.02	0.09	0.06	0.11*	0.12**	0.01	0.05
Insecure_t−1_- Insecure_t_	0.16**	0.13	−0.03	0.24**	0.18**	−0.06	0.03
Worried_t−1_- Insecure_t_	0.01	−0.01	−0.02	0.02	−0.02	−0.04	0.02
Cheerful_t−1_- Insecure_t_	0.10*	0.07	−0.03	−0.04	−0.01	0.03	−0.07
Down_t−1_- Worried_t_	0.17**	0.06	−0.11	0.04	0.09	0.06	−0.17
Agitated_t−1_- Worried_t_	0.03	0.15**	0.12	0.06	0.06	0.00	0.12
Insecure_t−1_- Worried_t_	−0.05	−0.03	0.03	0.01	0.05	0.04	−0.01
Worried_t−1_- Worried_t_	0.19**	0.07	−0.12	0.15**	0.12*	−0.03	−0.09
Cheerful_t−1_- Worried_t_	0.16**	0.07	−0.09	0.06	0.04	−0.02	−0.07
Down_t−1_ - Cheerful_t_	−0.08*	−0.01	0.07	−0.05	−0.16*	−0.11	0.18*
Agitated_t−1_- Cheerful_t_	0.04	−0.05	−0.09	−0.003	−0.04	−0.01	−0.08
Insecure_t−1_- Cheerful_t_	−0.02	−0.02	0.00	0.03	0.07	0.04	−0.04
Worried_t−1_- Cheerful_t_	0.00	−0.00	−0.01	−0.00	−0.01	−0.01	0.00
Cheerful_t−1_- Cheerful_t_	0.37**	0.42**	0.05	0.23**	0.38**	0.14	−0.09

Note: Base = baseline, Post = post-intervention, ∆ = change from baseline to post-intervention, differ = the difference in **Δ** between the imipramine and the placebo condition, the difference between the baseline and post-intervention coefficients do not always correspond with ∆ because only two decimals are shown (rounding differences), *p < 0.05, **p < 0.01.

**Table 3:** Mind-Maastricht Trial: Change in associations between mental states over time.

	MBCT			Waiting list control			Differ
	Base	Post	Δ	Base	Post	Δ	∆
Down_t−1_- Down_t_	0.17**	0.21**	0.04	0.19**	0.19**	−0.00	0.04
Agitated_t−1_- Down_t_	0.03	−0.02	−0.05	0.00	−0.01	−0.01	−0.04
Insecure_t−1_- Down_t_	0.05	0.06*	0.00	0.07*	0.02	−0.05	0.06
Worried_t−1_- Down_t_	0.08**	0.01	−0.07*	0.02	0.03	0.01	−0.08
Cheerful_t−1_- Down_t_	−0.10**	−0.07*	0.03	−0.14**	−0.11**	0.03	−0.00
Down_t−1_- Agitated_t_	0.03	−0.02	−0.05	0.01	−0.05	−0.07	0.01
Agitated_t−1_- Agitated_t_	0.28**	0.24**	−0.04	0.21**	0.21**	0.00	−0.04
Insecure_t−1_- Agitated_t_	0.03	0.02	−0.01	0.11**	0.02	−0.09	0.07
Worried_t−1_- Agitated_t_	0.04	0.02	−0.02	0.00	−0.01	−0.01	−0.01
Cheerful_t−1_- Agitated_t_	0.06	−0.03	−0.09	−0.05	−0.03	0.02	−0.11
Down_t−1_- Insecure_t_	0.09**	0.09*	−0.00	0.08**	0.01	−0.08*	0.07
Agitated_t−1_- Insecure_t_	0.03	0.06*	0.03	0.01	0.02	0.01	0.02
Insecure_t−1_- Insecure_t_	0.20**	0.17**	−0.03	0.21**	0.16**	−0.04	0.01
Worried_t−1_- Insecure_t_	0.07*	0.02	−0.04	0.03	0.03	0.00	−0.04
Cheerful_t−1_- Insecure_t_	−0.09**	−0.02	0.07	−0.05	−0.05	0.00	0.07
Down_t−1_- Worried_t_	0.05	0.03	−0.01	0.08*	0.03	−0.05	0.04
Agitated_t−1_- Worried_t_	0.03	0.01	−0.02	−0.01	−0.01	−0.00	−0.02
Insecure_t−1_- Worried_t_	0.09**	0.03	−0.06	0.07*	0.04	−0.04	−0.02
Worried_t−1_- Worried_t_	0.22**	0.22**	−0.01	0.17**	0.18**	0.01	−0.02
Cheerful_t−1_- Worried_t_	−0.11**	−0.06	0.05	−0.09**	−0.09**	0.00	0.04
Down_t−1_ - Cheerful_t_	−0.05	−0.13**	−0.08	−0.08*	−0.00	0.07	−0.15*
Agitated_t−1_- Cheerful_t_	−0.00	0.02	0.03	0.00	0.03	0.02	0.01
Insecure_t−1_- Cheerful_t_	−0.02	−0.01	0.01	−0.06*	−0.03	0.04	−0.02
Worried_t−1_- Cheerful_t_	−0.05	−0.02	0.03	0.01	−0.01	−0.03	0.06
Cheerful_t−1_- Cheerful_t_	0.27**	0.25**	−0.03	0.28**	0.29**	0.00	−0.03

Note: Base = baseline, Post = post-intervention, Δ = change from baseline to post-intervention, differ = the difference in Δ between the imipramine and the placebo condition, the difference between the baseline and post-intervention coefficients do not always correspond with **Δ** because only two decimals are shown (rounding differences), *p < 0.05, **p < 0.01.
