# Supplementary material for: The Impact of Treatments for Depression on the Dynamic Network Structure of Mental States: Two Randomized Controlled Trials
Source: Sci Rep. 2017 Apr 20;7:46523. doi: 10.1038/srep46523 (PMC5397847; doi:10.1038/srep46523)
Supplement: Supplementary Information [file srep46523-s1.pdf]

{1557244522614592538}

```
### Supplementary code for the paper:
###
### The impact of treatments for depression on the dynamic network structure of
mental states: Two randomized controlled trials.
### Evelien Snippe, Wolfgang Viechtbauer, Nicole Geschwind, Peter de Jonge, Marieke
Wichers
###
### Code for loading and preparing the data.
### Written by Wolfgang Viechtbauer.

############################################################################

### load data, sort data, and recode/rename variables so that the coding/naming is
consistent across trials
### group:  1 = ctrl, 2 = trt
### period: 1 = pre, 2 = post

### outcome names
outnames <- c("worry", "down", "cheerful", "agitated", "insecure")

if (study == "imi") {
   dat <- read.table("barge_ana_dev2_r.csv", sep=",", header=TRUE, na.strings="")
   dat <- dat[order(dat$subjno, dat$medgroup, dat$beepco_continuous),]
   dat$group  <- ifelse(dat$tca == 0, 1, 2)
   dat$period <- ifelse(dat$period == 1, 1, 2)
   names(dat)[pmatch(c("piekerde", "depresif", "opgewkt", "voegejaa", "onzeker"),
names(dat))] <- outnames
   names(dat)[pmatch(c("DevPM_piek_l", "DevPM_depr_l", "DevPM_opgew_l",
"DevPM_gejaa_l", "DevPM_onzek_l"), names(dat))] <- paste0(outnames, "_l")
}
if (study == "mbct") {
   dat <- read.table("mbct_prep_dev.csv", sep="\t", header=TRUE, na.strings="")
   dat <- dat[order(dat$subjno, dat$per_group, dat$beepco_continuous),]
   dat$group <- ifelse(dat$informat04 == 0, 1, 2)
   dat$period <- ifelse(dat$st_period == 2, 1, 2)
   names(dat)[pmatch(c("pieker", "somber__", "opgewkt_", "opgej", "onzeker_"),
names(dat))] <- outnames
   names(dat)[pmatch(c("DevPM_piek_l", "DevPM_somb_l", "DevPM_opgew_l",
"DevPM_opgej_l", "DevPM_onzek_l"), names(dat))] <- paste0(outnames, "_l")
}

############################################################################

### to avoid captured output being split across lines and to avoid scientific
notation
options(width=1000, scipen=100)

############################################################################




### Code for permutation testing of individual coefficients.
### Written by Wolfgang Viechtbauer.

############################################################################

rm(list=ls())
library(nlme)
library(parallel)

############################################################################
```

{1557244522614592538}

```
### select study to analyze
study <- "imi"
#study <- "mbct"

### number of permutation iterations
perms <- 100000

### number of cores available on computer
#detectCores()

### number of cores to use for (multicore) processing
ncpus <- 1

############################################################################

### load data, sort data, and recode/rename variables so that the coding/naming is
consistent across trials
### group:  1 = ctrl, 2 = trt
### period: 1 = pre, 2 = post

source("r_prep_data.r")

############################################################################

### permutation function

permfunc <- function(iter, dat) {

   library(nlme)

   ### reshuffle outcome variable within subjects (but 1st iteration is always the
original data)
   if (iter > 1)
      dat$outcome <- unlist(sapply(split(dat$outcome, dat$subjno), sample,
simplify=FALSE))

   ### fit model with reshuffled data
   res <- try(lme(outcome ~ worry_l + down_l + cheerful_l + agitated_l + insecure_l
+ beepco_continuous, random = ~ beepco_continuous | subjno, data=dat,
na.action=na.omit, control=list(opt="optim", msMaxIter=1000)), silent=TRUE)

   ### if model doesn't converge, return NA; otherwise return coefficients
   if (inherits(res, "try-error")) {
      return(NA)
   } else {
      return(fixef(res))
   }

}

############################################################################

### start local cluster for multicore processing
if (ncpus > 1)
   cl <- makePSOCKcluster(ncpus)

### set group and period numbers to loop through
grpnums <- 1:2
prdnums <- 1:2

for (grpnum in grpnums) {

   for (prdnum in prdnums) {
```

```
      for (outname in outnames) {

         cat("group:   ", grpnum, "\n")
         cat("period:  ", prdnum, "\n")
         cat("outcome: ", outname, "\n")

         dat.sel <- subset(dat, group == grpnum & period == prdnum)
         dat.sel$outcome <- dat.sel[,outname]

         ### fit model with actual data
         res <- lme(outcome ~ worry_l + down_l + cheerful_l + agitated_l + 
insecure_l + beepco_continuous, random = ~ beepco_continuous | subjno, data=dat.sel, 
na.action=na.omit, control=list(opt="optim", msMaxIter=1000))

         ### repeatedly apply permfunc() function
         time.start <- proc.time()
         if (ncpus == 1) {
            permres <- lapply(1:perms, permfunc, dat=dat.sel)
         } else {
            permres <- parLapply(cl, 1:perms, permfunc, dat=dat.sel)
         }
         time.end <- proc.time()
         cat("Minutes:", ((time.end - time.start)/60)[3], "\n")

         ### turn results into a matrix
         permres <- do.call(rbind, permres)

         ###################################################################

         ### table with model-based and permutation based p-values (two definitions 
of the p-values)
         p.perm.def1 <- p.perm.def2 <- rep(NA, length(fixef(res)))
         for (j in 1:length(fixef(res))) {
            p.perm.def1[j] <- 2*min(mean(permres[,j] >= fixef(res)[j], na.rm=TRUE), 
mean(permres[,j] <= fixef(res)[j], na.rm=TRUE))
            p.perm.def2[j] <- min(1, 2*ifelse(fixef(res)[j] > 0, mean(permres[,j] >= 
fixef(res)[j], na.rm=TRUE), mean(permres[,j] <= fixef(res)[j], na.rm=TRUE)))
         }

         ### save results to file
         sav <- round(cbind(summary(res)$tTable, "p-perm.def1"=p.perm.def1, 
"p-perm.def2"=p.perm.def2, "conv"=sum(!is.na(permres[,1]))), 6)
         capture.output(sav, file=paste0("results_", study, 
"/table_test_coefs_group_", grpnum, "_period_", prdnum, "_outcome_", outname, 
".txt"))

      }

   }

}

### stop local cluster for multicore processing
if (ncpus > 1)
   stopCluster(cl)

############################################################################




### Code for permutation testing of the change in individual coefficients.
```

```
### Written by Wolfgang Viechtbauer.

############################################################################

rm(list=ls())
library(nlme)
library(parallel)

############################################################################

### select study to analyze
study <- "imi"
#study <- "mbct"

### number of permutation iterations
perms <- 100000

### number of cores available on computer
#detectCores()

### number of cores to use for (multicore) processing
ncpus <- 1

############################################################################

### load data, sort data, and recode/rename variables so that the coding/naming is
consistent across trials
### group:  1 = ctrl, 2 = trt
### period: 1 = pre, 2 = post

source("r_prep_data.r")

############################################################################

### permutation function

permfunc <- function(iter, dat, nobs.per.person.P1, nobs.per.person.P2, n, outname)
{

   library(nlme)

   dat$outcome <- dat[,outname]

   ### reshuffle period variable within subjects (but 1st iteration is always the
original data)
   if (iter > 1)
      dat$period <- rep(replicate(n, sample(c(1,2))),
times=c(rbind(nobs.per.person.P1, nobs.per.person.P2)))

   ### fit models with reshuffled data
   res.P1 <- try(lme(outcome ~ worry_l + down_l + cheerful_l + agitated_l +
insecure_l + beepco_continuous, random = ~ beepco_continuous | subjno,
data=subset(dat, period==1), na.action=na.omit, control=list(opt="optim",
msMaxIter=1000)), silent=TRUE)
   res.P2 <- try(lme(outcome ~ worry_l + down_l + cheerful_l + agitated_l +
insecure_l + beepco_continuous, random = ~ beepco_continuous | subjno,
data=subset(dat, period==2), na.action=na.omit, control=list(opt="optim",
msMaxIter=1000)), silent=TRUE)

   ### if one of the models doesn't converge, return NA; otherwise return the change
in coefficients
   if (inherits(res.P1, "try-error") | inherits(res.P2, "try-error")) {
      return(NA)
```

{1557244522614592538}

```
   } else {
      return(fixef(res.P1) - fixef(res.P2))
   }

}

############################################################################

### start local cluster for multicore processing
if (ncpus > 1)
   cl <- makePSOCKcluster(ncpus)

### set group numbers to loop through
grpnums <- 1:2

for (grpnum in grpnums) {

   for (outname in outnames) {

      cat("grpnum:  ", grpnum, "\n")
      cat("outcome: ", outname, "\n")

      dat.sel <- subset(dat, group==grpnum)
      dat.sel$outcome <- dat.sel[,outname]

      dat.sel.P1 <- subset(dat.sel, period==1)
      dat.sel.P2 <- subset(dat.sel, period==2)

      ### fit models with actual data
      res.P1 <- try(lme(outcome ~ worry_l + down_l + cheerful_l + agitated_l +
insecure_l + beepco_continuous, random = ~ beepco_continuous | subjno,
data=dat.sel.P1, na.action=na.omit, control=list(opt="optim", msMaxIter=1000)),
silent=TRUE)
      res.P2 <- try(lme(outcome ~ worry_l + down_l + cheerful_l + agitated_l +
insecure_l + beepco_continuous, random = ~ beepco_continuous | subjno,
data=dat.sel.P2, na.action=na.omit, control=list(opt="optim", msMaxIter=1000)),
silent=TRUE)

      ### number of observations per person pre and post
      nobs.per.person.P1 <- sapply(split(dat.sel.P1$group, dat.sel.P1$subjno),
length)
      nobs.per.person.P2 <- sapply(split(dat.sel.P2$group, dat.sel.P2$subjno),
length)

      ### number of individuals (assumed to be the same at P1 and P2!)
      n <- length(nobs.per.person.P1)

      ### repeatedly apply permfunc() function
      time.start <- proc.time()
      if (ncpus == 1) {
         permres <- lapply(1:perms, permfunc, dat=dat.sel,
nobs.per.person.P1=nobs.per.person.P1, nobs.per.person.P2=nobs.per.person.P2, n=n,
outname=outname)
      } else {
         permres <- parLapply(cl, 1:perms, permfunc, dat=dat.sel,
nobs.per.person.P1=nobs.per.person.P1, nobs.per.person.P2=nobs.per.person.P2, n=n,
outname=outname)
      }
      time.end <- proc.time()
      cat("Minutes:", ((time.end - time.start)/60)[3], "\n")

      ### turn results into a matrix
      permres <- do.call(rbind, permres)
```

```
      ########################################################################

      ### table with permutation based p-values (two definitions of the p-values)
      b.diff.obs <- fixef(res.P2) - fixef(res.P1)
      p.perm.def1 <- p.perm.def2 <- rep(NA, length(b.diff.obs))
      for (j in 1:length(b.diff.obs)) {
         p.perm.def1[j] <- 2*min(mean(permres[,j] >= b.diff.obs[j], na.rm=TRUE),
mean(permres[,j] <= b.diff.obs[j], na.rm=TRUE))
         p.perm.def2[j] <- min(1, 2*ifelse(b.diff.obs[j] > 0, mean(permres[,j] >=
b.diff.obs[j], na.rm=TRUE), mean(permres[,j] <= b.diff.obs[j], na.rm=TRUE)))
      }

      ### save results to file
      sav <- round(cbind(b.diff.obs, "p-perm.def1"=p.perm.def1,
"p-perm.def2"=p.perm.def2, "conv"=sum(!is.na(permres[,1]))), 6)
      capture.output(sav, file=paste0("results_", study,
"/table_test_change_group_", grpnum, "_outcome_", outname, ".txt"))

   }

}

### stop local cluster for multicore processing
if (ncpus > 1)
   stopCluster(cl)

############################################################################



### Code for permutation testing of the difference in change in individual
coefficients.
### Written by Wolfgang Viechtbauer.

############################################################################

rm(list=ls())
library(nlme)
library(parallel)

############################################################################

### select study to analyze
study <- "imi"
#study <- "mbct"

### number of permutation iterations
perms <- 100000

### number of cores available on computer
#detectCores()

### number of cores to use for (multicore) processing
ncpus <- 1

############################################################################

### load data, sort data, and recode/rename variables so that the coding/naming is
consistent across trials
### group:  1 = ctrl, 2 = trt
### period: 1 = pre, 2 = post
```

```
source("r_prep_data.r")

############################################################################

### permutation function

permfunc <- function(iter, dat, nobs.per.person, group.per.person, outname) {

   library(nlme)

   dat$outcome <- dat[,outname]

   ### reshuffle group variable (but 1st iteration is always the original data)
   if (iter > 1)
      dat$group <- rep(sample(group.per.person), times=nobs.per.person)

   ### fit models with reshuffled data
   res.G1P1 <- try(lme(outcome ~ worry_l + down_l + cheerful_l + agitated_l + 
insecure_l + beepco_continuous, random = ~ beepco_continuous | subjno, 
data=subset(dat, group==1 & period==1), na.action=na.omit, control=list(opt="optim", 
msMaxIter=1000)), silent=TRUE)
   res.G1P2 <- try(lme(outcome ~ worry_l + down_l + cheerful_l + agitated_l + 
insecure_l + beepco_continuous, random = ~ beepco_continuous | subjno, 
data=subset(dat, group==1 & period==2), na.action=na.omit, control=list(opt="optim", 
msMaxIter=1000)), silent=TRUE)
   res.G2P1 <- try(lme(outcome ~ worry_l + down_l + cheerful_l + agitated_l + 
insecure_l + beepco_continuous, random = ~ beepco_continuous | subjno, 
data=subset(dat, group==2 & period==1), na.action=na.omit, control=list(opt="optim", 
msMaxIter=1000)), silent=TRUE)
   res.G2P2 <- try(lme(outcome ~ worry_l + down_l + cheerful_l + agitated_l + 
insecure_l + beepco_continuous, random = ~ beepco_continuous | subjno, 
data=subset(dat, group==2 & period==2), na.action=na.omit, control=list(opt="optim", 
msMaxIter=1000)), silent=TRUE)

   ### if one of the models doesn't converge, return NA; otherwise return the 
difference in the change in coefficients
   if (inherits(res.G1P1, "try-error") | inherits(res.G1P2, "try-error") | 
inherits(res.G2P1, "try-error") | inherits(res.G2P2, "try-error")) {
      return(NA)
   } else {
      return((fixef(res.G2P2) - fixef(res.G2P1)) - (fixef(res.G1P2) - 
fixef(res.G1P1)))
   }

}

############################################################################

### start local cluster for multicore processing
if (ncpus > 1)
   cl <- makePSOCKcluster(ncpus)

for (outname in outnames) {

   cat("outcome: ", outname, "\n")

   dat$outcome <- dat[,outname]

   ### fit models with actual data
   res.G1P1 <- try(lme(outcome ~ worry_l + down_l + cheerful_l + agitated_l + 
insecure_l + beepco_continuous, random = ~ beepco_continuous | subjno, 
data=subset(dat, group==1 & period==1), na.action=na.omit, control=list(opt="optim", 
msMaxIter=1000)), silent=TRUE)
```

```
   res.G1P2 <- try(lme(outcome ~ worry_l + down_l + cheerful_l + agitated_l +
insecure_l + beepco_continuous, random = ~ beepco_continuous | subjno,
data=subset(dat, group==1 & period==2), na.action=na.omit, control=list(opt="optim",
msMaxIter=1000)), silent=TRUE)
   res.G2P1 <- try(lme(outcome ~ worry_l + down_l + cheerful_l + agitated_l +
insecure_l + beepco_continuous, random = ~ beepco_continuous | subjno,
data=subset(dat, group==2 & period==1), na.action=na.omit, control=list(opt="optim",
msMaxIter=1000)), silent=TRUE)
   res.G2P2 <- try(lme(outcome ~ worry_l + down_l + cheerful_l + agitated_l +
insecure_l + beepco_continuous, random = ~ beepco_continuous | subjno,
data=subset(dat, group==2 & period==2), na.action=na.omit, control=list(opt="optim",
msMaxIter=1000)), silent=TRUE)

   ### number of observations per person (pre and post together)
   nobs.per.person <- sapply(split(dat$group, dat$subjno), length)

   ### group of each person (either control or treatment)
   group.per.person <- sapply(split(dat$group, dat$subjno), function(x) x[1])

   ### repeatedly apply permfunc() function
   time.start <- proc.time()
   if (ncpus == 1) {
      permres <- lapply(1:perms, permfunc, dat=dat, nobs.per.person=nobs.per.person,
group.per.person=group.per.person, outname=outname)
   } else {
      permres <- parLapply(cl, 1:perms, permfunc, dat=dat,
nobs.per.person=nobs.per.person, group.per.person=group.per.person, outname=outname)
   }
   time.end <- proc.time()
   cat("Minutes:", ((time.end - time.start)/60)[3], "\n")

   ### turn results into a matrix
   permres <- do.call(rbind, permres)

   ############################################################################

   ### table with permutation based p-values (two definitions of the p-values)
   b.diff.obs <- (fixef(res.G2P2) - fixef(res.G2P1)) - (fixef(res.G1P2) -
fixef(res.G1P1))
   p.perm.def1 <- p.perm.def2 <- rep(NA, length(b.diff.obs))
   for (j in 1:length(b.diff.obs)) {
      p.perm.def1[j] <- 2*min(mean(permres[,j] >= b.diff.obs[j], na.rm=TRUE),
mean(permres[,j] <= b.diff.obs[j], na.rm=TRUE))
      p.perm.def2[j] <- min(1, 2*ifelse(b.diff.obs[j] > 0, mean(permres[,j] >=
b.diff.obs[j], na.rm=TRUE), mean(permres[,j] <= b.diff.obs[j], na.rm=TRUE)))
   }

   ### save results to file
   sav <- round(cbind(b.diff.obs, "p-perm.def1"=p.perm.def1,
"p-perm.def2"=p.perm.def2, "conv"=sum(!is.na(permres[,1]))), 6)
   capture.output(sav, file=paste0("results_", study, "/table_test_diff_outcome_",
outname, ".txt"))

}

### stop local cluster for multicore processing
if (ncpus > 1)
   stopCluster(cl)

############################################################################
```

{15572445226145925381}

```
### Code for permutation testing of change in mean absolute connection strength.
### Written by Wolfgang Viechtbauer.

############################################################################

rm(list=ls())
library(nlme)
library(parallel)

############################################################################

### select study to analyze
study <- "imi"
#study <- "mbct"

### number of permutation iterations
perms <- 100000

### number of cores available on computer
#detectCores()

### number of cores to use for (multicore) processing
ncpus <- 1

############################################################################

### load data, sort data, and recode/rename variables so that the coding/naming is
consistent across trials
### group:  1 = ctrl, 2 = trt
### period: 1 = pre, 2 = post

source("r_prep_data.r")

############################################################################

### permutation function

permfunc <- function(iter, dat, nobs.per.person.P1, nobs.per.person.P2, n, outnames)
{

   library(nlme)

   ### reshuffle period variable within subjects (but 1st iteration is always the
original data)
   if (iter > 1)
      dat$period <- rep(replicate(n, sample(c(1,2))),
times=c(rbind(nobs.per.person.P1, nobs.per.person.P2)))

   ### matrices for storing the coefficients
   b.P1 <- matrix(NA, nrow=length(outnames), ncol=length(outnames))
   b.P2 <- matrix(NA, nrow=length(outnames), ncol=length(outnames))

   for (outname in outnames) {

      dat$outcome <- dat[,outname]

      ### fit models with reshuffled data
      res.P1 <- try(lme(outcome ~ worry_l + down_l + cheerful_l + agitated_l +
insecure_l + beepco_continuous, random = ~ beepco_continuous | subjno,
data=subset(dat, period==1), na.action=na.omit, control=list(opt="optim",
msMaxIter=1000)), silent=TRUE)
      res.P2 <- try(lme(outcome ~ worry_l + down_l + cheerful_l + agitated_l +
insecure_l + beepco_continuous, random = ~ beepco_continuous | subjno,
```

```
data=subset(dat, period==2), na.action=na.omit, control=list(opt="optim",
msMaxIter=1000)), silent=TRUE)

      ### if one of the models doesn't converge, break out of loop
      if (inherits(res.P1, "try-error") | inherits(res.P2, "try-error"))
         break

      ### store coefficients
      b.P1[,which(outname == outnames)] <- fixef(res.P1)[2:(length(outnames)+1)]
      b.P2[,which(outname == outnames)] <- fixef(res.P2)[2:(length(outnames)+1)]

   }

   ### if one of the coefficients is NA, return NA; otherwise return the change in
mean absolute connection strength
   if (any(is.na(b.P1)) | any(is.na(b.P2))) {
      return(NA)
   } else {
      return(mean(abs(b.P2)) - mean(abs(b.P1)))
   }

}

############################################################################

### start local cluster for multicore processing
if (ncpus > 1)
   cl <- makePSOCKcluster(ncpus)

### set group numbers to loop through
grpnums <- 1:2

### matrices for storing the coefficients
b.P1 <- matrix(NA, nrow=length(outnames), ncol=length(outnames),
dimnames=list(outnames,outnames))
b.P2 <- matrix(NA, nrow=length(outnames), ncol=length(outnames),
dimnames=list(outnames,outnames))

for (grpnum in grpnums) {

   for (outname in outnames) {

      cat("grpnum:  ", grpnum, "\n")
      cat("outcome: ", outname, "\n")

      dat.sel <- subset(dat, group==grpnum)
      dat.sel$outcome <- dat.sel[,outname]

      dat.sel.P1 <- subset(dat.sel, period==1)
      dat.sel.P2 <- subset(dat.sel, period==2)

      ### fit models with actual data
      res.P1 <- try(lme(outcome ~ worry_l + down_l + cheerful_l + agitated_l +
insecure_l + beepco_continuous, random = ~ beepco_continuous | subjno,
data=dat.sel.P1, na.action=na.omit, control=list(opt="optim", msMaxIter=1000)),
silent=TRUE)
      res.P2 <- try(lme(outcome ~ worry_l + down_l + cheerful_l + agitated_l +
insecure_l + beepco_continuous, random = ~ beepco_continuous | subjno,
data=dat.sel.P2, na.action=na.omit, control=list(opt="optim", msMaxIter=1000)),
silent=TRUE)

      ### store coefficients
      b.P1[,which(outname == outnames)] <- fixef(res.P1)[2:(length(outnames)+1)]
```

```
      b.P2[,which(outname == outnames)] <- fixef(res.P2)[2:(length(outnames)+1)]

   }

   ### number of observations per person pre and post
   nobs.per.person.P1 <- sapply(split(dat.sel.P1$group, dat.sel.P1$subjno), length)
   nobs.per.person.P2 <- sapply(split(dat.sel.P2$group, dat.sel.P2$subjno), length)

   ### number of individuals (assumed to be the same at P1 and P2!)
   n <- length(nobs.per.person.P1)

   ### repeatedly apply permfunc() function
   time.start <- proc.time()
   if (ncpus == 1) {
      permres <- lapply(1:perms, permfunc, dat=dat.sel,
nobs.per.person.P1=nobs.per.person.P1, nobs.per.person.P2=nobs.per.person.P2, n=n,
outnames=outnames)
   } else {
      permres <- parLapply(cl, 1:perms, permfunc, dat=dat.sel,
nobs.per.person.P1=nobs.per.person.P1, nobs.per.person.P2=nobs.per.person.P2, n=n,
outnames=outnames)
   }
   time.end <- proc.time()
   cat("Minutes:", ((time.end - time.start)/60)[3], "\n")

   ### turn results into a matrix
   permres <- do.call(rbind, permres)

   ############################################################################

   ### table with permutation based p-values (two definitions of the p-values)
   b.diff.obs <- mean(abs(b.P2)) - mean(abs(b.P1))
   p.perm.def1 <- 2*min(mean(permres >= b.diff.obs, na.rm=TRUE), mean(permres <=
b.diff.obs, na.rm=TRUE))
   p.perm.def2 <- min(1, 2*ifelse(b.diff.obs > 0, mean(permres >= b.diff.obs,
na.rm=TRUE), mean(permres <= b.diff.obs, na.rm=TRUE)))

   ### save results to file
   sav <- round(cbind(b.diff.obs, "p-perm.def1"=p.perm.def1,
"p-perm.def2"=p.perm.def2, "conv"=sum(!is.na(permres[,1]))), 6)
   capture.output(sav, file=paste0("results_", study, "/table_test_change_group_",
grpnum, "_total.txt"))

}

### stop local cluster for multicore processing
if (ncpus > 1)
   stopCluster(cl)

############################################################################



### Code for permutation testing of the difference in change in mean absolute
connection strength.
### Written by Wolfgang Viechtbauer.

############################################################################

rm(list=ls())
library(nlme)
library(parallel)
```

```
################################################################################

### select study to analyze
study <- "imi"
#study <- "mbct"

### number of permutation iterations
perms <- 100000

### number of cores available on computer
#detectCores()

### number of cores to use for (multicore) processing
ncpus <- 1

################################################################################

### load data, sort data, and recode/rename variables so that the coding/naming is
consistent across trials
### group:  1 = ctrl, 2 = trt
### period: 1 = pre, 2 = post

source("r_prep_data.r")

################################################################################

### permutation function

permfunc <- function(iter, dat, nobs.per.person, group.per.person, outnames) {

   library(nlme)

   ### reshuffle group variable (but 1st iteration is always the original data)
   if (iter > 1)
      dat$group <- rep(sample(group.per.person), times=nobs.per.person)

   ### matrices for storing the coefficients
   b.G1P1 <- matrix(NA, nrow=length(outnames), ncol=length(outnames),
dimnames=list(outnames,outnames))
   b.G1P2 <- matrix(NA, nrow=length(outnames), ncol=length(outnames),
dimnames=list(outnames,outnames))
   b.G2P1 <- matrix(NA, nrow=length(outnames), ncol=length(outnames),
dimnames=list(outnames,outnames))
   b.G2P2 <- matrix(NA, nrow=length(outnames), ncol=length(outnames),
dimnames=list(outnames,outnames))

   for (outname in outnames) {

      dat$outcome <- dat[,outname]

      ### fit models with reshuffled data
      res.G1P1 <- try(lme(outcome ~ worry_l + down_l + cheerful_l + agitated_l +
insecure_l + beepco_continuous, random = ~ beepco_continuous | subjno,
data=subset(dat, group==1 & period==1), na.action=na.omit, control=list(opt="optim",
msMaxIter=1000)), silent=TRUE)
      res.G1P2 <- try(lme(outcome ~ worry_l + down_l + cheerful_l + agitated_l +
insecure_l + beepco_continuous, random = ~ beepco_continuous | subjno,
data=subset(dat, group==1 & period==2), na.action=na.omit, control=list(opt="optim",
msMaxIter=1000)), silent=TRUE)
      res.G2P1 <- try(lme(outcome ~ worry_l + down_l + cheerful_l + agitated_l +
insecure_l + beepco_continuous, random = ~ beepco_continuous | subjno,
data=subset(dat, group==2 & period==1), na.action=na.omit, control=list(opt="optim",
msMaxIter=1000)), silent=TRUE)
```

```
      res.G2P2 <- try(lme(outcome ~ worry_l + down_l + cheerful_l + agitated_l +
insecure_l + beepco_continuous, random = ~ beepco_continuous | subjno,
data=subset(dat, group==2 & period==2), na.action=na.omit, control=list(opt="optim",
msMaxIter=1000)), silent=TRUE)

      ### if one of the models doesn't converge, break out of loop
      if (inherits(res.G1P1, "try-error") | inherits(res.G1P2, "try-error") |
inherits(res.G2P1, "try-error") | inherits(res.G2P2, "try-error"))
         break

      ### store coefficients
      b.G1P1[,which(outname == outnames)] <- fixef(res.G1P1)[2:(length(outnames)+1)]
      b.G1P2[,which(outname == outnames)] <- fixef(res.G1P2)[2:(length(outnames)+1)]
      b.G2P1[,which(outname == outnames)] <- fixef(res.G2P1)[2:(length(outnames)+1)]
      b.G2P2[,which(outname == outnames)] <- fixef(res.G2P2)[2:(length(outnames)+1)]

   }

   ### if one of the coefficients is NA, return NA; otherwise return the difference
in the change in mean absolute connection strength
   if (any(is.na(b.G1P1)) | any(is.na(b.G1P2)) | any(is.na(b.G2P1)) |
any(is.na(b.G2P2))) {
      return(NA)
   } else {
      return((mean(abs(b.G2P2)) - mean(abs(b.G2P1))) - (mean(abs(b.G1P2)) -
mean(abs(b.G1P1))))
   }

}

############################################################################

### start local cluster for multicore processing
if (ncpus > 1)
   cl <- makePSOCKcluster(ncpus)

### matrices for storing the coefficients
b.G1P1 <- matrix(NA, nrow=length(outnames), ncol=length(outnames),
dimnames=list(outnames,outnames))
b.G1P2 <- matrix(NA, nrow=length(outnames), ncol=length(outnames),
dimnames=list(outnames,outnames))
b.G2P1 <- matrix(NA, nrow=length(outnames), ncol=length(outnames),
dimnames=list(outnames,outnames))
b.G2P2 <- matrix(NA, nrow=length(outnames), ncol=length(outnames),
dimnames=list(outnames,outnames))

for (outname in outnames) {

   cat("outcome: ", outname, "\n")

   dat$outcome <- dat[,outname]

   ### fit models with actual data
   res.G1P1 <- try(lme(outcome ~ worry_l + down_l + cheerful_l + agitated_l +
insecure_l + beepco_continuous, random = ~ beepco_continuous | subjno,
data=subset(dat, group==1 & period==1), na.action=na.omit, control=list(opt="optim",
msMaxIter=1000)), silent=TRUE)
   res.G1P2 <- try(lme(outcome ~ worry_l + down_l + cheerful_l + agitated_l +
insecure_l + beepco_continuous, random = ~ beepco_continuous | subjno,
data=subset(dat, group==1 & period==2), na.action=na.omit, control=list(opt="optim",
msMaxIter=1000)), silent=TRUE)
   res.G2P1 <- try(lme(outcome ~ worry_l + down_l + cheerful_l + agitated_l +
insecure_l + beepco_continuous, random = ~ beepco_continuous | subjno,
```

```
data=subset(dat, group==2 & period==1), na.action=na.omit, control=list(opt="optim",
msMaxIter=1000)), silent=TRUE)
   res.G2P2 <- try(lme(outcome ~ worry_l + down_l + cheerful_l + agitated_l +
insecure_l + beepco_continuous, random = ~ beepco_continuous | subjno,
data=subset(dat, group==2 & period==2), na.action=na.omit, control=list(opt="optim",
msMaxIter=1000)), silent=TRUE)

   ### store coefficients
   b.G1P1[,which(outname == outnames)] <- fixef(res.G1P1)[2:(length(outnames)+1)]
   b.G1P2[,which(outname == outnames)] <- fixef(res.G1P2)[2:(length(outnames)+1)]
   b.G2P1[,which(outname == outnames)] <- fixef(res.G2P1)[2:(length(outnames)+1)]
   b.G2P2[,which(outname == outnames)] <- fixef(res.G2P2)[2:(length(outnames)+1)]

}

### number of observations per person (pre and post together)
nobs.per.person <- sapply(split(dat$group, dat$subjno), length)

### group of each person (either control or treatment)
group.per.person <- sapply(split(dat$group, dat$subjno), function(x) x[1])

### repeatedly apply permfunc() function
time.start <- proc.time()
if (ncpus == 1) {
   permres <- lapply(1:perms, permfunc, dat=dat, nobs.per.person=nobs.per.person,
group.per.person=group.per.person, outnames=outnames)
} else {
   permres <- parLapply(cl, 1:perms, permfunc, dat=dat,
nobs.per.person=nobs.per.person, group.per.person=group.per.person,
outnames=outnames)
}
time.end <- proc.time()
cat("Minutes:", ((time.end - time.start)/60)[3], "\n")

### turn results into a matrix
permres <- do.call(rbind, permres)

############################################################################

### table with permutation based p-values (two definitions of the p-values)
b.diff.obs <- (mean(abs(b.G2P2)) - mean(abs(b.G2P1))) - (mean(abs(b.G1P2)) -
mean(abs(b.G1P1)))
p.perm.def1 <- 2*min(mean(permres >= b.diff.obs, na.rm=TRUE), mean(permres <=
b.diff.obs, na.rm=TRUE))
p.perm.def2 <- min(1, 2*ifelse(b.diff.obs > 0, mean(permres >= b.diff.obs,
na.rm=TRUE), mean(permres <= b.diff.obs, na.rm=TRUE)))

### save results to file
sav <- round(cbind(b.diff.obs, "p-perm.def1"=p.perm.def1, "p-perm.def2"=p.perm.def2,
"conv"=sum(!is.na(permres[,1]))), 6)
capture.output(sav, file=paste0("results_", study, "/table_test_diff_total.txt"))

### stop local cluster for multicore processing
if (ncpus > 1)
   stopCluster(cl)

############################################################################
```

{1557244522614592538}
